# Drug repurposing to target Ebola virus replication and virulence using structural systems pharmacology

**DOI:** 10.1186/s12859-016-0941-9

**Published:** 2016-02-18

**Authors:** Zheng Zhao, Che Martin, Raymond Fan, Philip E. Bourne, Lei Xie

**Affiliations:** High Magnetic Field Laboratory, Chinese Academy of Sciences, Hefei, P. R. China; National Center for Biotechnology Information, National Library of Medicine, National Institutes of Health, Bethesda, MD USA; The Graduate Center, The City University of New York, New York, USA; Department of Chemistry, Hunter College, The City University of New York, New York, USA; Office of the Director, National Institutes of Health, Bethesda, MD USA; Department of Computer Science, Hunter College, The City University of New York, New York, USA

**Keywords:** Drug repositioning, Infectious disease, Indinavir, Sinefungin, Binding site similarity, RNA-directed RNA polymerase, VP24

## Abstract

**Background:**

The recent outbreak of Ebola has been cited as the largest in history. Despite this global health crisis, few drugs are available to efficiently treat Ebola infections. Drug repurposing provides a potentially efficient solution to accelerating the development of therapeutic approaches in response to Ebola outbreak. To identify such candidates, we use an integrated structural systems pharmacology pipeline which combines proteome-scale ligand binding site comparison, protein-ligand docking, and Molecular Dynamics (MD) simulation.

**Results:**

One thousand seven hundred and sixty-six FDA-approved drugs and 259 experimental drugs were screened to identify those with the potential to inhibit the replication and virulence of Ebola, and to determine the binding modes with their respective targets. Initial screening has identified a number of promising hits. Notably, Indinavir; an HIV protease inhibitor, may be effective in reducing the virulence of Ebola. Additionally, an antifungal (Sinefungin) and several anti-viral drugs (e.g. Maraviroc, Abacavir, Telbivudine, and Cidofovir) may inhibit Ebola RNA-directed RNA polymerase through targeting the MTase domain.

**Conclusions:**

Identification of safe drug candidates is a crucial first step toward the determination of timely and effective therapeutic approaches to address and mitigate the impact of the Ebola global crisis and future outbreaks of pathogenic diseases. Further in vitro and in vivo testing to evaluate the anti-Ebola activity of these drugs is warranted.

**Electronic supplementary material:**

The online version of this article (doi:10.1186/s12859-016-0941-9) contains supplementary material, which is available to authorized users.

## Background

The recent Ebola outbreak poses a serious threat to human health around the world and has been cited as the largest Ebola outbreak in history [1]. Efficient therapeutics with the ability to cure Ebola infections are yet to be available. Despite recent technological advances, the conventional drug discovery and development process often takes more than 10 years, and costs more than 2 billion dollars to bring a new drug to market [2]. New approaches are urgently needed to deliver medicines to treat Ebola in a timely fashion.

Repurposing safe drugs to be anti-infectious agents has emerged as a novel concept to combat pathogens, and to accelerate drug development [3–7], especially given that the ADME and toxicology properties of approved drugs are already known. Moreover, computational approaches provide an attractive solution in determining potential drug repurposing opportunities, especially where in vitro and/or in vivo screening is difficult or even impossible [8]. It should be noted, however, that several unique challenges are encountered during *in silico* anti-infective drug repurposing. For example, the successful phenotype-based method [9] which compares molecular or organismal phenotypes of drug response with those of diseases, has limitations when applied to anti-infective drug development. Notably, it is not trivial to compare drug response and disease phenotype across human and pathogens. Additionally, ligand- and target-based drug repurposing are limited by their under-representative coverage of drug targets in the pathogen genomes [10]. Finally, few virulence-related proteins have characterized ligands, even though their structures are readily available [7].

Previously, we developed a structural systems pharmacology approach, to identify drug-target interactions on a proteome scale by integrating proteome-wide ligand binding site comparison [11, 12], protein-ligand docking [13], and Molecular Dynamics (MD) simulation with systems biology modeling [7, 11, 14–21]. Here, we apply this proven successful strategy to reveal FDA-approved and experimental drugs with the potential to inhibit the replication and virulence of Ebola. Here we focused on two main Ebola targets: RNA-directed RNA polymerase (L) and VP24 [22, 23]. RNA polymerase plays a key role in RNA transcription and replication [22]. Thus, the inhibition of RNA polymerase in Ebola may inhibit its replication. Ebola VP24 interacts with human Karyopherin alpha to disarm the human immune system [24, 25]. Thus, the inhibition of VP24 may disrupt the VP24-Karyopherin alpha interaction and reduce the virulence of Ebola. The 3D structure of RNA polymerase was obtained by homology modeling while the druggable binding site of VP24 was explored using MD simulations. The MD simulation has made significant contributions in structure-based drug design in recent years [18, 26–34]. The MD simulation allows us not only to investigate conformational flexibility which plays an important role in molecular recognition, [30] but also to reveal the potential druggable binding site on the receptor that is not evident from static X-ray structures [18, 26–29, 31, 32]. One thousand seven hundred sixty-six FDA-approved drugs and 259 nucleotide/nucleoside experimental drugs in DrugBank [35] have been computationally screened against these two targets. As there is not a single docking program performed well for all targets [36], we used multiple docking software packages to obtain the consensus results to avoid the bias of some docking tools. Our initial screening has identified several promising hits. Specifically, Indinavir, an HIV protease inhibitor, may also reduce the virulence of Ebola based on it high binding affinity to VP24. Additionally, the antifungal drug Sinfungin may inhibit Ebola RNA-directed RNA polymerase through targeting its MTase domain. The detailed binding modes of these promising hits with their respective targets have been determined. The results presented here can be used as a stepping stone to validate the anti-Ebola activity of these drugs through both in vivo and in vitro experimentation, and hence may offer new opportunities to design efficient anti-Ebola therapeutics.

## Methods

### Structural systems pharmacology pipeline

**Fig. 1:**
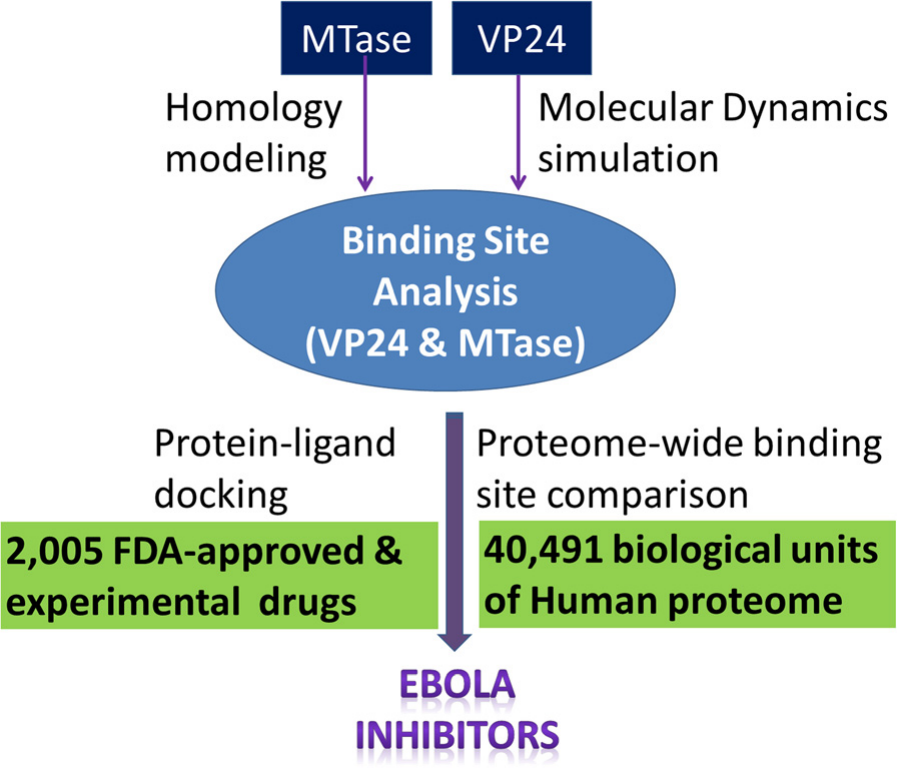
The pipeline of structural systems pharmacology approach in this study

The structural systems pharmacology approach has been successfully applied to the prediction of side effect [15, 37], drug repurposing [10, 14, 38], polypharmacology drug design [16-18, 39], and other applications [12, 20, 40, 41]. Here we used the strategy to determine effective drugs which target Ebola virus. A summary of the protocol is shown in Fig. 1. Compounds from our drug library were screened based on two targets VP24 and MTase. The binding pocket of VP24 was obtained based on the trajectory from VP24 molecule dynamics simulation. The structure of MTase was built by homology model, and verified by the model evaluation software, Verify3D [42, 43] and PROCHECK [44]. Binding site similarity between the targets and the structural proteome was determined by SMAP [11, 12, 40]. Finally, candidate inhibitors were selected based on the consensus docking scores from multi-docking packages and dock pose analysis.

### Ligand binding site comparison on a structural proteome scale

Forty thousand four hundred and ninety-one biological units of solved complex structures that are co-crystallized with small molecules with at least five carbon atoms are compared with the predicted binding site of VP24, and SAM co-factor and substrate binding sites in MTase of RdRp using the ligand binding site comparison software SMAP [11, 12, 40]. Top ranked binding sites with their co-crystallized ligands with a *p*-value < 0.05 are subject to further analysis.

### Homology modeling

A homology model of O’-2-MTase was constructed iteratively to optimize its binding site conformation. First, human CAP-specific mRNA 2′-O-MTase (PDB id: 4N49) was used as a template to build a homology model whose secondary structure fragments surrounding the SAM binding site are optimized, i.e., no atomic crashes with the putative SAM conformation derived from SMAP. Modeller v9.14 [45] was used in this step. Second, I-TASSER that can build a model from multiple targets was used to add and optimize other fragments: ARG1-ALA26 and SER59-GLU87 to the model. Finally, loops were further optimized using Modeller v9.14. The final model was verified by Verify3D, which determines the compatibility of an atomic model (3D) with its own amino acid sequence (1D) [42, 43], and PROCHECK, which is a program to check the stereochemical quality of protein structures [44].

### Protein-ligand docking

The 3D coordinates of 1766 FDA-approved, DrugBank [35] annotated, non-redundant drugs were downloaded from ZINC database. Additionally, given that a number of nucleotide/nucleoside drugs demonstrated anti-viral properties, 259 nucleotide/nucleoside experimental drugs in DrugBank (updated 2014.03.19) were included in our drug candidates. These drugs were docked to VP24 and RNA-directed RNA polymerase using four docking packages Audodock4 [46], Autodock Vina [47], PLANTS [48], and Surflex [49]. Virtual screening analysis via the AutoDockTools 4 used the following settings in addition to the default docking parameters: ga_num_evals = 1750000, ga_pop_size = 150, ga_run = 20, and rmstol = 2.0. The top confirmation and score for interesting results were output. In Autodock Vina, the research space was redefined by the center coordinate and the size of every dimension of the grid box. The top 1 conformation and score were output. In Surflex, the proto was first obtained by predicting the binding site and protomol. The default parameters are set. The top 1 conformation and score were output. In PLANTS, to dock the drug lib, the screen mode is chosen, the binding site center is redefined as is done for Autodock Vina and the binding site radius is set as 12.5 Å. The top 1 conformation and score were output.

### Consensus scoring of protein-ligand docking

For all drug molecules, the corresponding docking scores from each docking tool were ranked. The score correlation was analyzed between two different docking tools by a linear fit. The correlation coefficient is 0.20, 0.43, and 0.61 between the Autodock Vina and Surflex scores, between the Autodock Vina and the PLANTS scores, and between the Surflex and the PLANTS scores, respectively, as shown in Additional file 1: Figure S1. As the correlation is higher between the Surflex and PLANTS scores, the top scored compounds from Surflex and PLANTS were further prioritized. If a drug was ranked within the top 100 by both Surflex and PLANTS, it was selected for further analysis.

### MD simulation

We performed a MD simulation to investigate the conformation change of VP24 protein in water. The simulation system was set up using Xleap based on the PDB id: 4M0Q. The TIP3P water box was added with a minimal wall distance of 12.0 Å from the VP24 and 11,237 water molecules were included. The simulation was performed using ACEMD. VP24 was described using the AMBER99SB force field on an NVIDIA GPU machine [50, 51]. The other parameters for the MD simulation were set at 300 K and 1.0 bar and with a 12.0 Å cutoff for the non-bonded interactions. The time step was 4 fs with the SHAKE algorithm [52]. A 200 ns equilibration protocol had been employed, and the trajectory was analyzed using the ptraj plugin. The system reached an equilibration state after 10 ns and the RMSD of the trajectory was shown in Additional file 1: Figure S2. From the equilibrated trajectory, the conformations were clustered based on RMSD. The binding pocket was predicted by Surflex [49] for the representative conformation of every cluster. The volumes of the binding pocket were determined by CASTp [53]. Finally, the conformation with the largest pocket was chosen.

## Results and discussion

### Drugs that may disrupt Ebola-human interaction

**Fig. 2:**
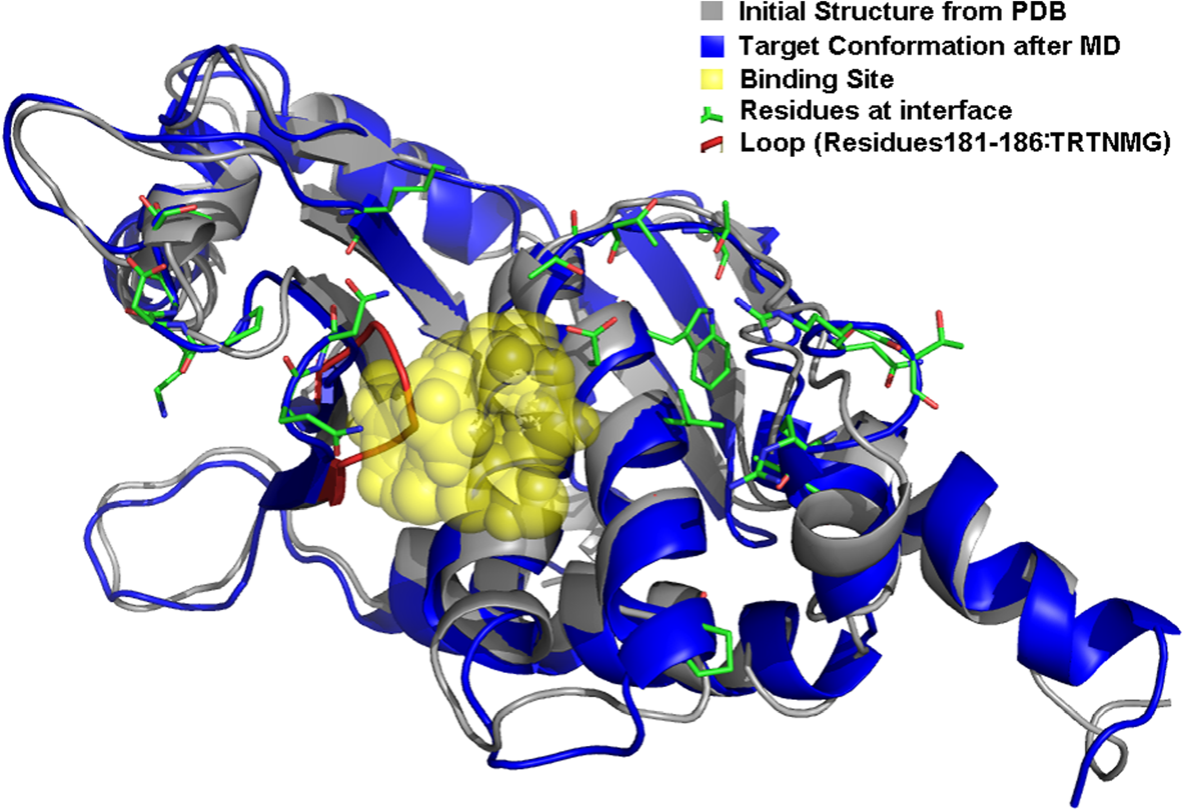
The binding interface of VP24 of Ebola with Karyopherin alpha. Interface residues are shown as stick models. An open pocket is shown as transparent yellow spheres. Initial conformation from PDB and conformation generated from MD simulation is shown in grey and blue, respectively. The loop (amino acids 181–186) that has a prominent conformational change after MD simulation is shown in red

The VP24 protein which is responsible for the Ebola-Human interaction has a solved PDB structure (PDB id: 4M0Q). While there is no known pocket that can accommodate a small molecule in its protein-protein interaction (PPI) interface with human Karyopherin alpha, a small molecule binding site in the PPI interface can be formed through conformation selection [54]. We applied MD simulations to obtain a sample of the conformation of VP24. A 200 ns MD was carried out using ACEMD on the GPU machine [51]. The largest pocket was formed after a 12 ns simulation and was located in the VP24-Karyopherin alpha binding interface (Fig. 2, amino acids on the interface are depicted in sticks while the binding site is in transparent yellow). As a comparison, the initial structure from PDB is showed in gray. Adjacent to the binding site, the loop (red color, amino acids 181–186) has a prominent conformational change during MD simulation, but remains at the interface of the PPI. Small molecule binding may interfere with the PPI at this VP24-Karyopherin alpha interface, thus leading to the interruption of the host-virus interaction, and inhibition of the virus [55]. The conformation of VP24 after the 12 ns simulation was subsequently used to screen potential inhibitors of VP24.

**Fig. 3:**
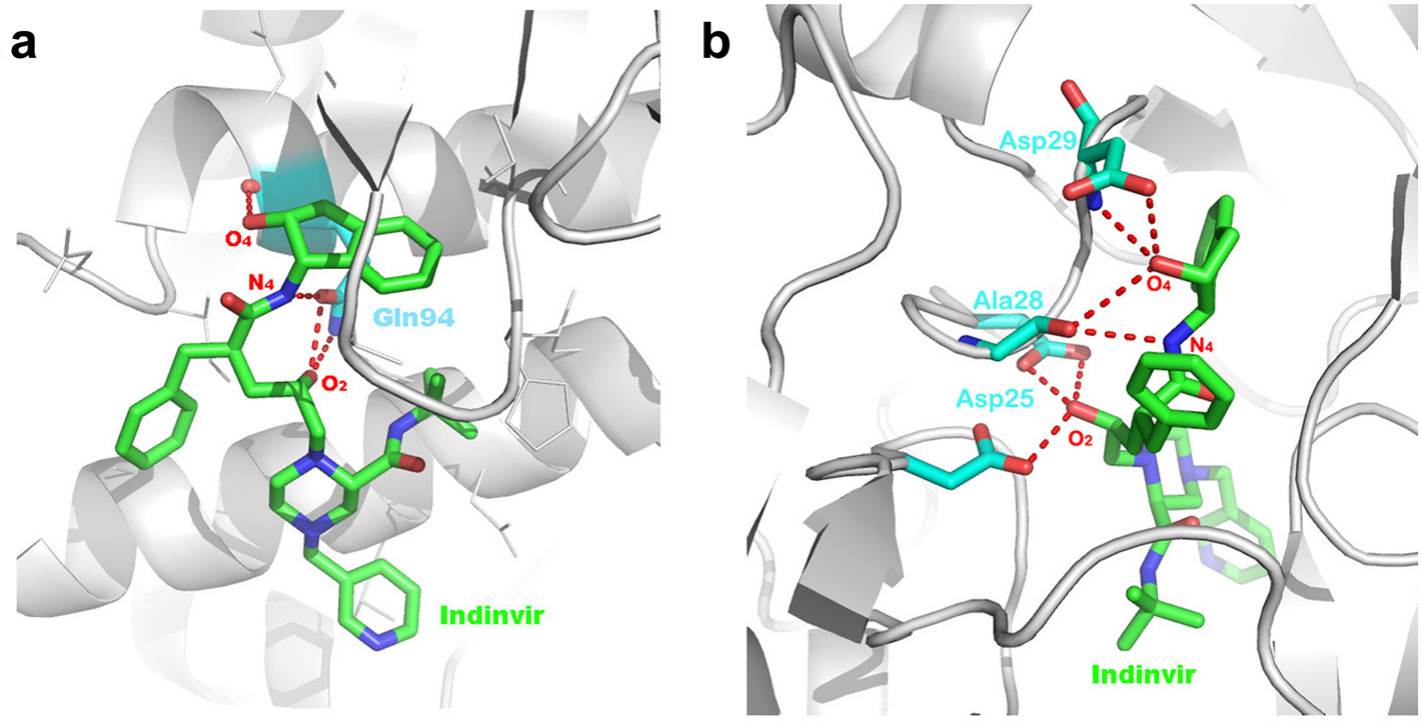
**a** The predicted binding mode of Indinavir in VP24 of Ebola (**a**) and (**b**) the binding mode of Indinavir in HIV protease (PDB id 2AVO)

To identify existing drugs which may inhibit VP24, a search for proteins with binding sites similar to that of the VP24 PPI interface was conducted. Here, proteome-wide ligand binding site comparison was carried out using SMAP [11, 12, 50]. The rationale is that similar binding sites may bind to similar molecules. The binding site of HIV protease was identified to be the most similar to that of VP24 (SMAP *p*-value < 0.05). Furthermore, 1766 FDA-approved drugs were docked to the VP24 binding pocket using multiple protein-ligand docking tools. Potential binders were ranked by their consensus (see method section for details). Consistent with the result from the ligand binding site similarity search, Indinavir: a HIV protease inhibitor, was ranked second in the protein-ligand docking study (Table 1). Its binding mode illustrated by Pymol [56] and Ligplot + [57] is shown in the Additional file 1: Figures S1b and S4b. Additional file 1: Figure S4b shows that the binding pocket of VP24 readily accommodates Indinavir depicting three hydrogen bonds between Indinavir and VP24. Notably, 11 amino acids form hydrophobic interactions with Indinavir, as showed in Additional file 1: Figure S3b. As illustrated in Fig. 3a, three hydrogen-bonding interactions exist between Indinavir and VP24: i) the O_2_ atom of Indinavir and the nitrogen atom from the sidechain of residue Gln94 in VP24, ii) the atom N_4_ of Indinavir and the oxygen atom in the sidechain of VP24’s Gln94, and iii) the O_4_ atom of Indinavir and the oxygen atom from the main chain of Gln94 of VP24. Figure 3b which illustrates Indinavir bound to its primary target. Moreover, we also compared the binding modes of Indinavir in its primary target, HIV protease, to its predicted Ebola target VP24, Fig. 3. Here, the Indinavir-HIV complex was downloaded from Protein Data Bank (PDB id 2AVO) [58]. Interestingly, HIV protease, reveals that the same atoms (O_2_, N_4_ and O_4_) of Indinavir form the hydrogen bonds with residues; Ala28, Asp29, Asp25 in Chain A and Asp25 in Chain B of HIV protease. Consequently, the predicted similar binding pattern of VP24 and HIV protease to Indinavir suggest that this HIV protease inhibitor may be repurposed to target Ebola VP24.

**Table 1:** Putative inhibitors of VP24, along with their structures, docking scores from four docking software packages, and primary targets

Drug name	Structure	Docking score				Primary target
		Surflex	Plants	Vina	Auto dock	
Montelukast		8.6	−102.97	−6.6	−5.11	Human leukotriene receptor
Indinavir		8.2	−98.66	−6.5	−6.98	HIV protease
Iloprost		7.5	−97.91	−6.1	−7.24	Human Prostacyclin receptor
hSalmeterol Xinafoate		7.0	−95.99	−5.3	−4.59	Human beta-2 adrenergic receptor
Travoprost		7.2	−95.92	−6.5	−5.21	Human prosaglandin F2-alpha receptor
Latanoprost		7.8	−95.68	−6.1	−5.61	Human prosaglandin F2-alpha receptor
Remikiren		7.3	−95.29	−6.7	−4.59	Human renin
Vitamin K1		7.2	−92.93	−6.1	−6.34	Human Vitamin K-dependent carboxylase
Mitoxantrone		9.9	−92.07	−6.0	−7.42	Human DNA topoisomerase 2α
Labetalol hydrochloride		7.7	−90.8	−6.0	−6.36	Human 1,1,2 adrenergic receptor
Tafluprost		8.1	−90.7	−5.9	−5.36	Human prosaglandin F2-alpha receptor
Misoprostol		7.1	−89.75	−5.4	−4.09	Human prostaglandin E2 receptor
Carboprost		7.3	−89.6	−5.5	−5.18	Human prostaglandin E2 receptor
Fosinopril		6.9	−88.92	−6.8	−6.85	Human angiotension-converting enzyme
Benzylpenicilloyl Polylysine		6.9	−88.71	−6.4	−6.34	Human immunoglobulin receptor
Bimatoprost		6.8	−88.37	−6.0	−5.82	Human prosaglandin F2-receptor
Nebivolol		7.2	−88.06	−5.6	−8.13	Human beta-1 adrenergic receptor
Valrubicin		6.8	−87.08	−7.1	−7.32	Human DNA topoisomerase 2α
Tamsulosin		6.8	−87.02	−6.3	−6.45	Human Alpha-1A adrenergic receptor
Mycophenolate Mofetil		7.4	−86.87	−5.9	−6.04	Human Inosine-5’-monophosphate dehydrogenase

In addition to HIV protease inhibitors, the top 20 ranked drugs; Table 1, (binding modes illustrated in Additional file 1: Figures S3 and S4), are enriched by GPCR-targeted drugs, especially for adrenergic receptors and prostaglandin receptors (*p*-value = 1.4e-4). Most of these drugs are administered for ocular hypertension or hypertension. Notably, they may serve as additional lead compounds towards the design of selective inhibitors of VP24. Interestingly, like the binding mode of Indinavir in VP24, the binding modes of the other 19 inhibitors, show conserved electrostatic interactions between the respective drug and VP24’s Gln94; shown in Additional file 1: Figure S3a, c, f, i, j, k, n, o, t. It should be noted, in some of the presented drug-target interactions, there are electrostatic interactions from other VP24 amino acids such as: Asp95 for drug hSaleterol Xinafoate (Additional file 1: Figure S3d), Asp115 for drug Tafluprost (Additional file 1: Figure S3k), and Asp99 for drug Benzylpenicilloyl Polylysine (Additional file 1: Figure S3o). Another major residue Gly173 also provide the main hydrogen-bonding interactions for the corresponding drugs as showed in Additional file 1: Figure S3d, e, g, j, m, p–t. Here again, we observe some hydrogen-bonding interactions from other amino acids such as: Ile172 for drug Iloprost (Additional file 1: Figure S3c), Ile98 for drug Remikiren (Additional file 1: Figure S3g), Gly111 for drug Mitoxantrone (Additional file 1: Figure S3i), His177 for drug Misoprostol (Additional file 1: Figure S3l), Thr174 for drug Fosinopril (Additional file 1: Figure S3n), Ile98 for drug Benzylpenicilloyl Polylysine (Additional file 1: Figure S3o), Ile172 for drug Bimatoprost (Additional file 1: Figure S3p), Ile98 for drug Valrubicin (Additional file 1: Figure S3r) and Gln175 for Mycophenolate Mofetil (Additional file 1: Figure S3t).

Importantly, hydrophobic interactions also contribute to the drug-target associations presented in the current study. Specifically, the binding pocket consists of about ten amino acids which form a hydrophobic environment; illustrated by spoked arcs and residue name. Further exploration of these binding modes may provide crucial information towards the design of lead compounds targeting VP24.

### Inhibitors of SAM-dependent 2′-O-MTase domain of RNA-directed RNA polymerase

The RNA-directed RNA polymerase L of Ebola was predicted to contain four major domains using an HHPred alignment [59] by searching against Pfam library. The aligned domains included: i) a Mononegavirals RdRp-like catalytic domain (residues 10–1090), ii) a mRNA-capping region V domain (residue 1104–1309), iii) a mRNA Guanine-7-MTase (residue 1472–1850), and iv) a SAM-dependent 2′-O-Methyltransferase (MTase) domain (residues 1804–2006), as shown in Table 2. Notably, no structures of these domains have been experimentally solved, and with the exception of 2′-O-MTase domain, no reliable structural template and alignment can be detected for the other three domains. A reliable homology model of 2′-O-MTase was constructed and verified by Verify3d and PROCHECK. Notably, the Verify3D shows 83.33 % of the residues had an averaged 3D-1D score > = 0.2; the PROCHECK suggests that 80 % residues in the most favorable regions, as shown in Additional file 1: Figure S5. More importantly, scores for the residues composing binding pocket was relatively higher (red color in Additional file 1: Figure S5a), and these residues fell into the allowed regions in the Ramachandran plot (Additional file 1: Figure S5b). These results suggest that our model is suitable for further docking studies. The homology model was subsequently applied to screen for potential competitive inhibitors of the 2′-O-MTase binding site.

**Table 2:** Putative Pfam domains of Ebola RNA-directed RNA polymerase L, along with their annotations, e-value of HHPred alignment, and start and end position of the alignment

Pfam family	Annotation	E-value	Start position	End position
PF00946	Mononegavirals RdRp	6.0e–212	10	1090
PF14318	mRNAcapping region V	1.2e–57	1104	1359
PF12803	G–7-MTase	7.5e–46	1472	1850
PF14314	2′-O-MTase	1.4e–13	1804	2006

Here, a scan was conducted across 40,491biological units of PDB structures to identify ligand binding sites similar to those present in the modeled 2′-O-MTase using SMAP. Consequently, it is not surprising that SAM binding pockets of multiple MTase were aligned with the 2′-O-MTase model with high statistical significance (*p*-value < 1.0e–3). The binding pose of SAM in the 2′-O-MTase model was determined by the superimposition of the modeled 2′-O-MTase binding site unto that of the most similar structure; human CAP-specific mRNA 2′-O-MTase from (PDB id: 4 N49). In addition to SAM which is known ligand of MTase, an antifungal drug Sinefungin was identified with high statistical significance (*p*-value = 1.8e–3).

Protein-ligand docking experiments were conducted to further verify predictions from the ligand binding site comparison. In addition to the ligands determined by SMAP, a number of SAM analogs (e.g. A9145C and aza-S-adenosyl-L-methionine) and anti-virus drugs were included in the screening. Because SAM is the known ligand of 2′-O-MTase, it is assumed that true binders of 2′-O-MTase should be ranked higher than or close to SAM. Among the identified putative inhibitors of 2′-O-MTase, several consistently ranked at the top or higher than SAM by four docking software packages: Surflex, PLANTS, Autodock Vina and Autodock, Table 3. Notably, the RMSD between the redocked SAM molecule and the conformation inferred from solved structure by SMAP was 0.964 Ǻ; suggesting reliable docking results.

**Table 3:** Putative inhibitors of SAM binding site of 2′-O-MTase, along with their structures, docking scores from four docking software packages, and primary targets

Compound	Structure	Docking score				Primary target
		Surflex	PLANTS	Vina	Auto Dock	
SAM		10.7	−109.03	−7.5	−7.34	Glycine N-methyltransferase
aza-S-adenosyl-L-methionine		9.3	−104.34	−8.9	−8.67	Glycine N-methyltransferase
Sinefungin		8.5	−103.68	−7.9	−9.59	Glycine N-methyltransferase
A9145C		7.0	−82.20	−6.3	−11.09	Glycine N-methyltransferase
Maraviroc		8.1	−98.7	−8.3	−10.94	C-C chemokine receptor type 5
Abacavir		7.2	−73.9	−6.8	−6.27	nucleoside analog reverse transcriptase inhibitor
Telbivudine		5.7	−74.4	−6.4	−5.77	Protein P
Cidofovir		6.7	−78.3	−7.2	−5.57	DNA polymerase catalytic subunit

**Fig. 4:**
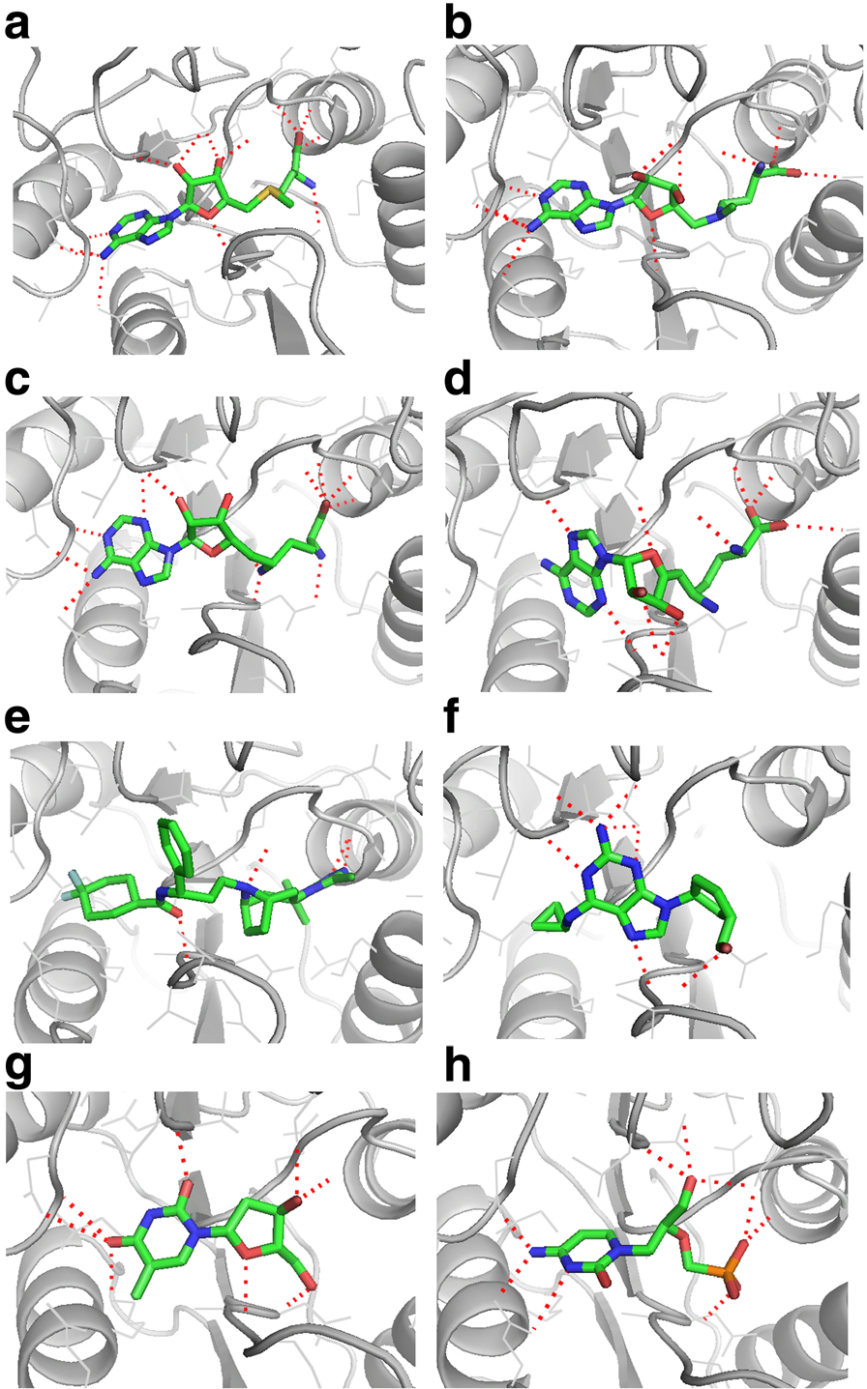
The predicted binding mode of drugs that are listed in Table 3 in 2′-O-MTase. The chemical compounds in the panels are: **a** SAM, **b** aza-S-adenosyl-L-methionine, **c** Sinefungin, **d** A9145C, **e** Maraviroc, **f** Abacavir, **g** Telbivudine, and **h** Cidofovir

Here, Sinefungin and A9145C are antiviral, antifungal, and antibacterial agents, whose structures are analogous to SAM [56, 60–66]. It is well known that aza-S-adenosyl-L-methionine inhibits mRNA cap methyltransferase [67]. Maraviroc is a chemokine receptor antagonist that is designed to act against HIV by interfering with the interaction between HIV and CCR5 [68]. Abacavir is a powerful nucleoside analog reverse transcriptase inhibitor against HIV [69]. Telbivudine is a synthetic thymidine nucleoside analog with specific activity against the hepatitis B virus [70]. Cidofovir is an antiviral medication for the treatment of cytomegalovirus (CMV) retinitis [71]. Notably, our results reveal that for the first time, the possible molecular mechanism of drug action for, Cidofovir. These finding suggest that Cidofovir may have activity against the Ebola virus and may additionally provide critical insight into the design of more potent and selective anti-Ebola therapeutic agents. Figure 4 and Additional file 1: Figure S6 show the putative binding mode of these inhibitors in 2′-O-MTase. Multiple hydrogen bonds form between Cidofovir and 2′-O-MTase including Leu54, Ser58 and Glu137. For the other 7 drugs, the binding modes also shows that amino acids Gla34, Ala35, Gly36, Leu54, Ser58, Asp99 and Ile100 are key residues in hydrogen-bonding interactions. Notably, amino acids within the binding pocket also provide conserved hydrophobic interactions; illustrated in Additional file 1: Figure S6 using the spoked arcs and residue names.

In the current study, a single conformation of the receptor structure was used for compound screening using protein-ligand docking. The bias in the scoring functions was minimized by using multiple types of docking software [72] including Surflex, PLANTS, AutoDock and Autodock Vina. Ensemble docking; a powerful approach which use multiple conformations and is widely used in virtual screening [73–75] allows for flexibility in protein receptors. In the presented high-throughput protocol however, docking is used to identify the initial promising hits. Hence, the priori verification of sampling accuracy will be limited [76]. Moreover, the choice of score function also affects the (ensemble) docking performance. Due to a lack of known reference ligands, it is difficult to determine which conformation ensemble and scoring function are optimal. We will apply the ensemble approach in the near future as more protein-ligand interaction and mutagenesis data become available.

## Conclusion

In the current study, we incorporated a proven structural systems pharmacology approach to identify several existing anti-virus and anti-fungal drugs which may be able to target and inhibit critical biological processes such as virus replication and virulence in Ebola. Collectively, in addition to identifying a number of lead compounds which may aid in the design of VP24 inhibitors, our analysis revealed two very promising drug candidates for repurposing: Sinfungin which may inhibit Ebola’s RNA polymerase activity, and Indinavir which may possibly disrupt Ebola-human interactions. Although further in vitro and in vivo experiments are needed to validate these *in silico* predictions, identification of these candidates is a crucial first step toward the determination of timely and effective therapeutic approaches to address and mitigate the impact of the Ebola global crisis and future outbreaks of pathogenic diseases.

## Additional file

Additional file 1: Figure S1. The correlations of docking scores between Surflex and Vina, between PLANTS and Vina, and between PLANTS and Surflex, respectively. **Figure S2.** The 200ns MD trajectory of VP24. **Figure S3.** The binding mode of the top 20 ranked drugs against VP24. (a) Montelukast, (b) Indinavir, (c) Iloprost, (d) hSalmeterol Xinafoate, (e) Travoprost, (f) Latanoprost, (g) Remikiren, (h) Vitamin K1, (i) Mitoxantrone, (j) Labetalol hydrochloride, (k) Tafluprost, (l) Misoprostol, (m) Carboprost, (n) Fosinopril, (o) Benzylpenicilloyl Polylysine, (p) Bimatoprost, (q) Nebivolol, (r) Valrubicin, (s) Tamsulosin, (t) Mycophenolate Mofetil. **Figure S4.** The 3D binding mode of the top 20 ranked drugs on the target of VP24. (a) Montelukast, (b) Indinavir, (c) Iloprost, (d) hSalmeterol Xinafoate, (e) Travoprost, (f) Latanoprost, (g) Remikiren, (h) Vitamin K1, (i) Mitoxantrone, (j) Labetalol hydrochloride, (k) Tafluprost, (l) Misoprostol, (m) Carboprost, (n) Fosinopril, (o) Benzylpenicilloyl Polylysine, (p) Bimatoprost, (q) Nebivolol, (r) Valrubicin, (s) Tamsulosin, (t) Mycophenolate Mofetil. **Figure S5.** Structural quality assessment of the homology model of O’-2-MTase. (a) Verify3D score. The sphere in red color showed the residues of composing binding site. (b) Ramachandran Plot using PROCHECK. **Figure S6.** The predicted binding mode of drugs that are listed in Table 3 in 2’-O-MTase. The drugs in the panels are: (a) SAM, (b) aza-S-adenosyl-L-methionine, (c) Sinefungin, (d) A9145C, (e) Maraviroc, (f) Abacavir, (g) Telbivudine, and (h) Cidofovir. (DOCX 3178 kb)
