# Supplementary material for: Drug repurposing to target Ebola virus replication and virulence using structural systems pharmacology
Source: BMC Bioinformatics. 2016 Feb 18;17:90. doi: 10.1186/s12859-016-0941-9 (PMC4757998; doi:10.1186/s12859-016-0941-9)

**Drug Repurposing to Target Ebola Virus Replication and Virulence Using Structural Systems Pharmacology**

Zheng Zhao^1,2^, Che Martin^3^, Raymond Fan^4^, Philip E. Bourne^5^ , Lei Xie^3,6,*^

^1^High Magnetic Field Laboratory, Chinese Academy of Sciences, P. R. China

^2^National Center for Biotechnology Information, National Library of Medicine, National Institute of Health, Bethesda, MD, U.S.A

^3^The Graduate Center, The City University of New York, U. S. A.

^4^Department of Chemistry, Hunter College, The City University of New York, U. S. A.

^5^Office of the Director, National Institutes of Health, Bethesda, MD, U. S. A.

^6^Department of Computer Science, Hunter College, The City University of New York, U. S. A.

*To whom correspondence should be addressed

[lei.xie@hunter.cuny.edu](mailto:lei.xie@hunter.cuny.edu) (LX)

**Supplemental Materials**

**S_Fig. 1.** The correlations of docking scores between Surflex and Vina, between PLANTS and Vina, and between PLANTS and Surflex, respectively

**
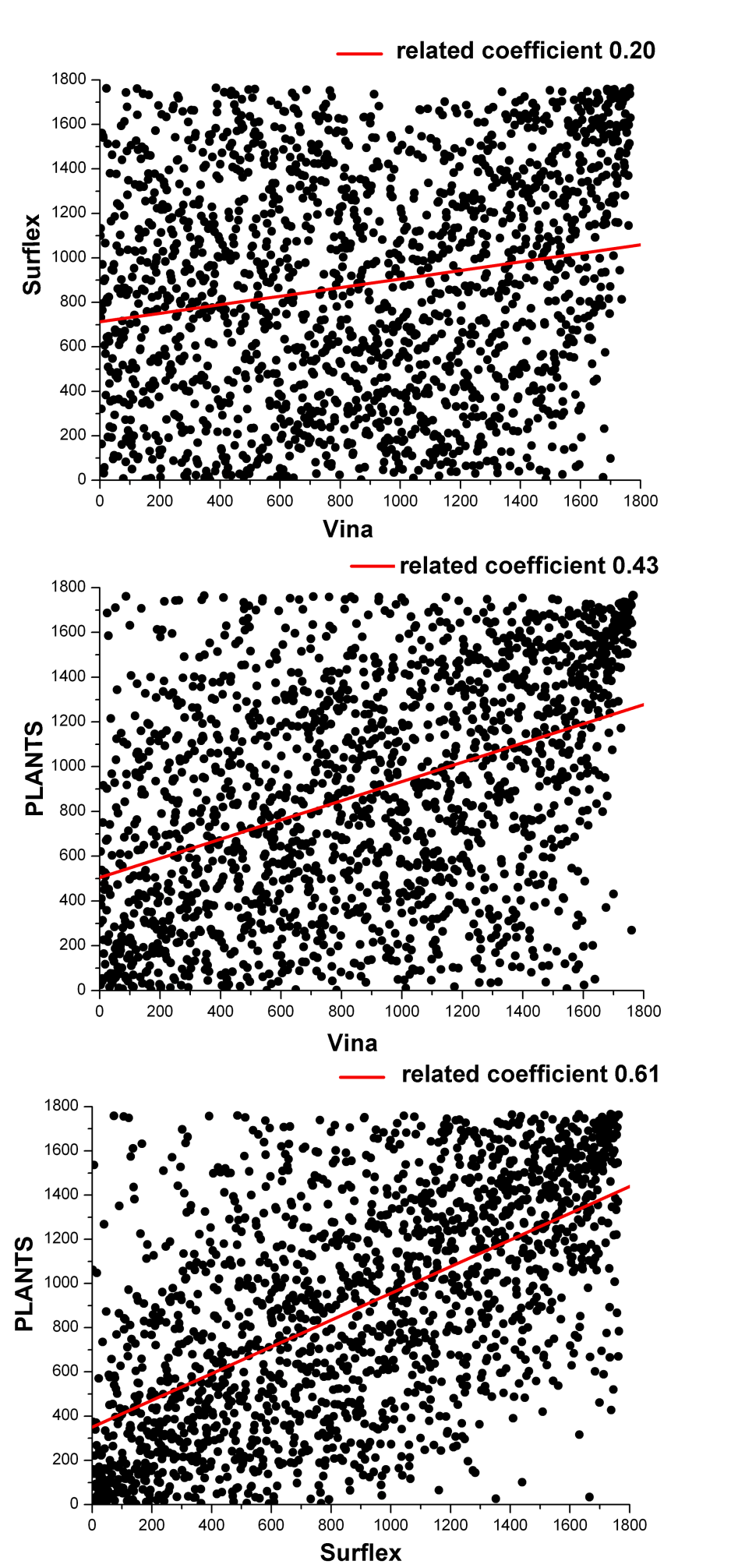
 S_Fig. 2.** The 200ns MD trajectory of VP24.





**S_Fig.3.** The binding mode of the top 20 ranked drugs against VP24. (a) Montelukast, (b) Indinavir, (c) Iloprost, (d) hSalmeterol Xinafoate, (e) Travoprost, (f) Latanoprost, (g) Remikiren, (h) Vitamin K1, (i) Mitoxantrone, (j) Labetalol hydrochloride, (k) Tafluprost, (l) Misoprostol, (m) Carboprost, (n) Fosinopril, (o) Benzylpenicilloyl Polylysine, (p) Bimatoprost, (q) Nebivolol, (r) Valrubicin, (s) Tamsulosin, (t) Mycophenolate Mofetil


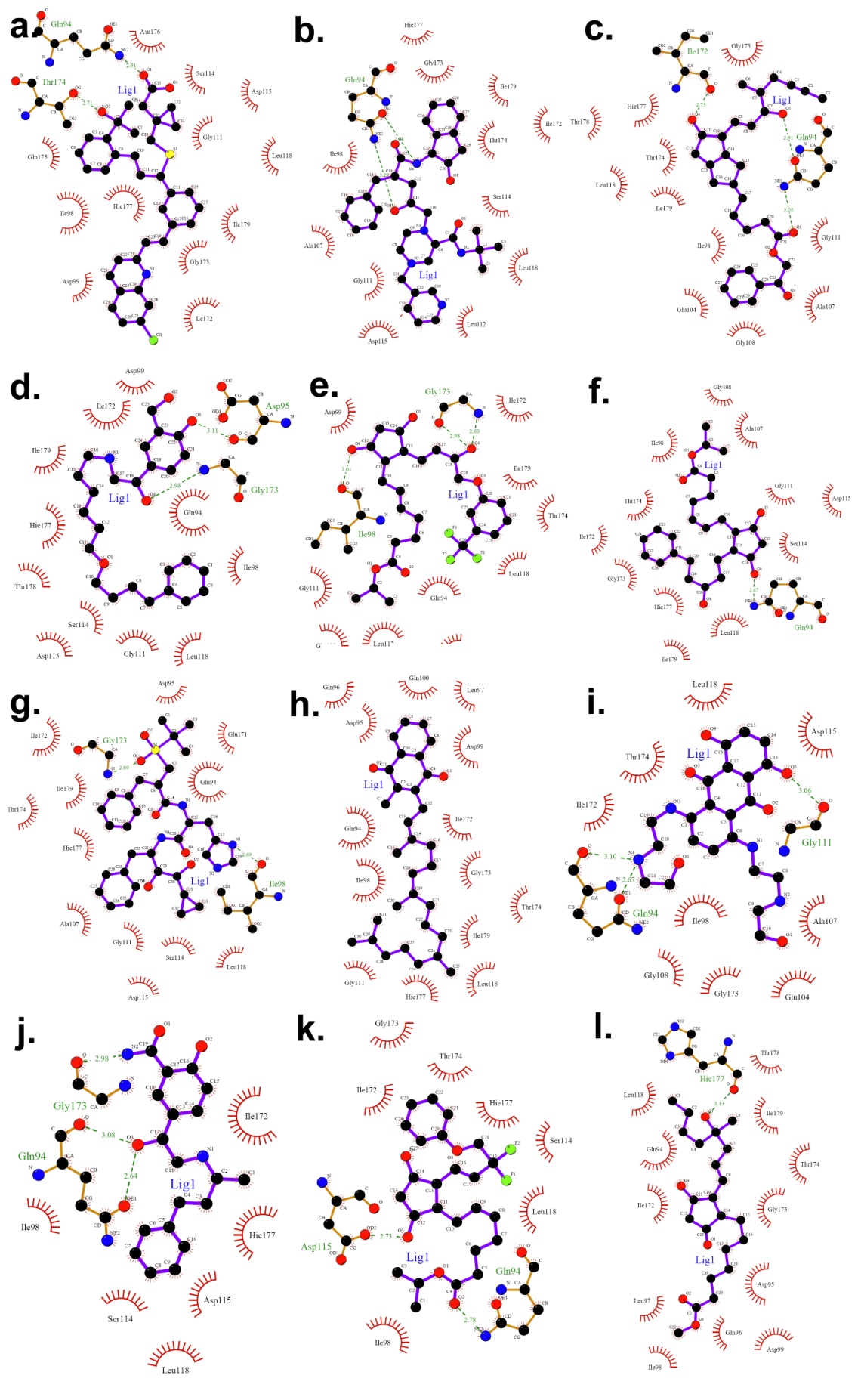

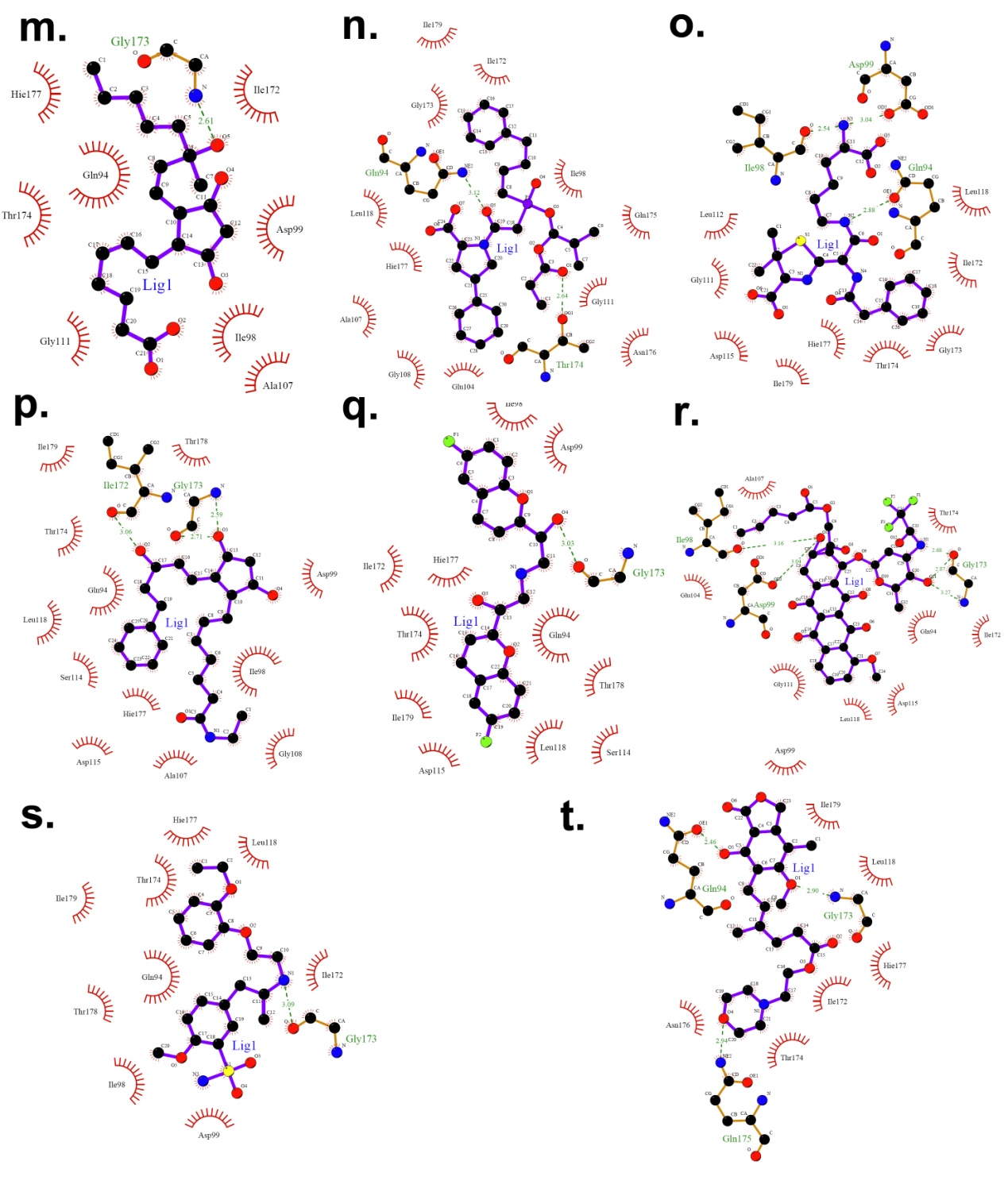


**S_Fig.4.** The 3D binding mode of the top 20 ranked drugs on the target of VP24. (a) Montelukast, (b) Indinavir, (c) Iloprost, (d) hSalmeterol Xinafoate, (e) Travoprost, (f) Latanoprost, (g) Remikiren, (h) Vitamin K1, (i) Mitoxantrone, (j) Labetalol hydrochloride, (k) Tafluprost, (l) Misoprostol, (m) Carboprost, (n) Fosinopril, (o) Benzylpenicilloyl Polylysine, (p) Bimatoprost, (q) Nebivolol, (r) Valrubicin, (s) Tamsulosin, (t) Mycophenolate Mofetil


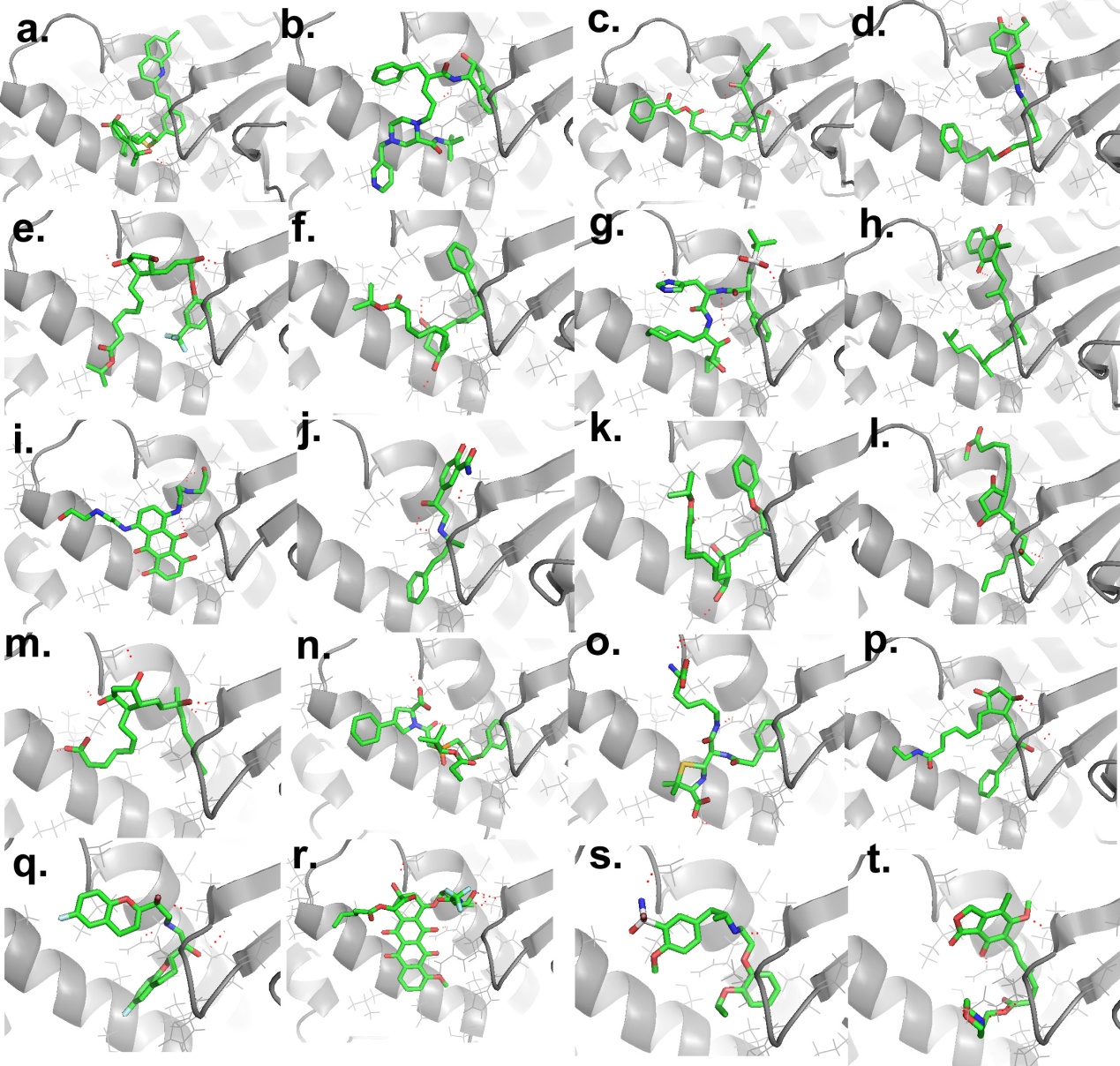


**S_Fig.5.** Structural quality assessment of the homology model of O’-2-MTase. (a) Verify3D score. The sphere in red color showed the residues of composing binding site. (b) Ramachandran Plot using PROCHECK.


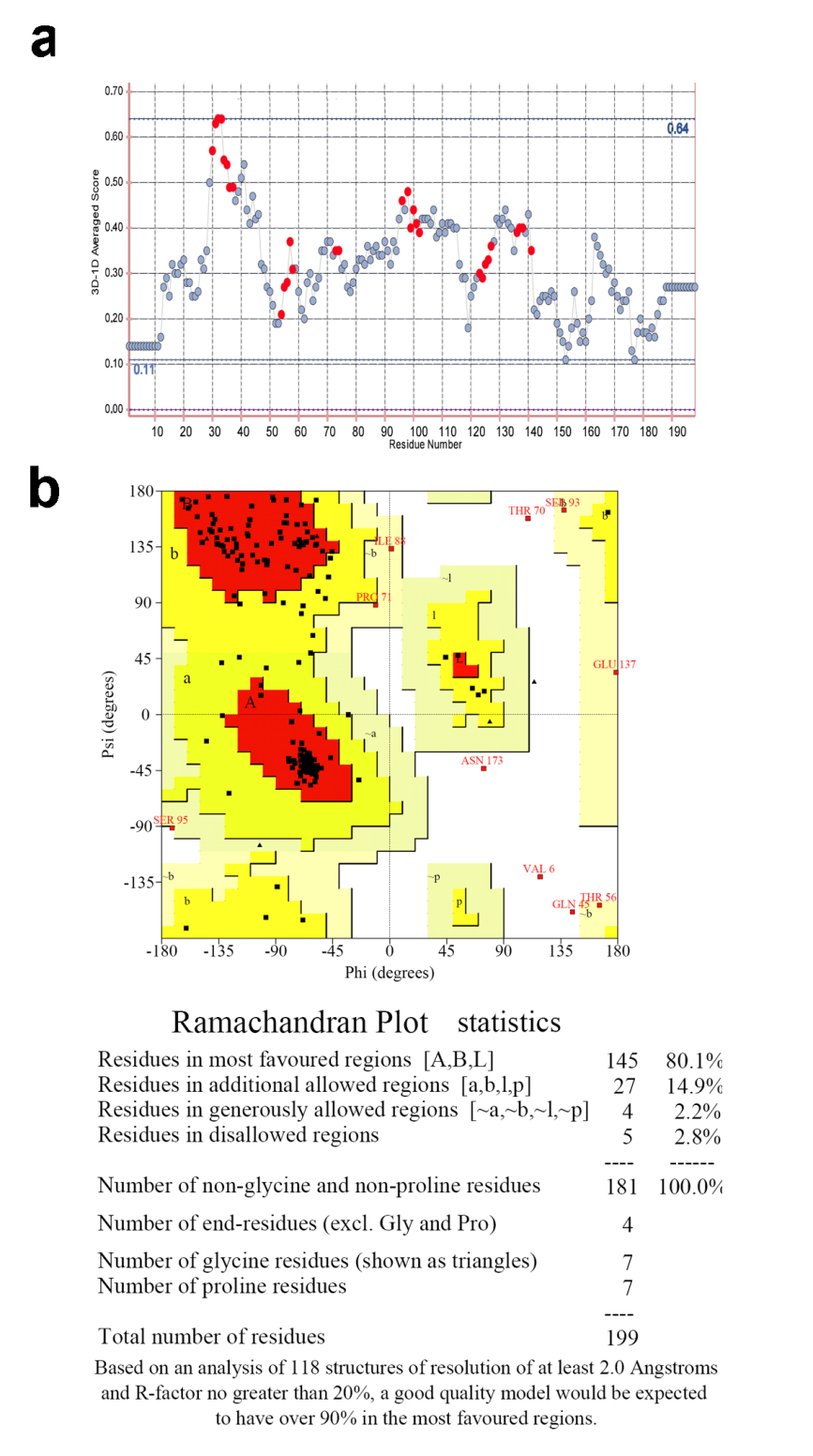


**S_Fig. 6**. The predicted binding mode of drugs that are listed in Table 3 in 2’-O-MTase. The drugs in the panels are: (a) SAM, (b) aza-S-adenosyl-L-methionine, (c) Sinefungin, (d) A9145C, (e) Maraviroc, (f) Abacavir, (g) Telbivudine, and (h) Cidofovir.


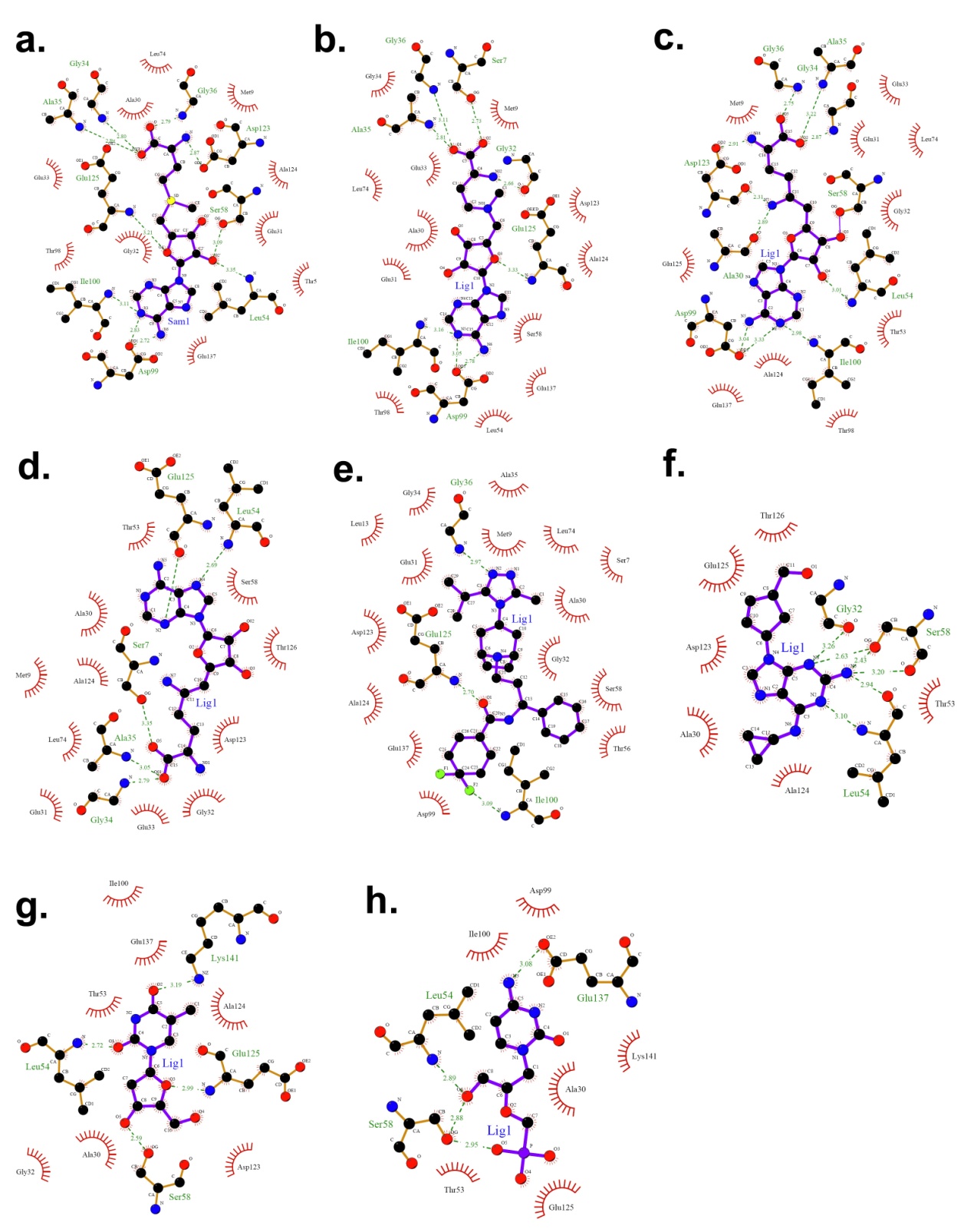

Supplement: Additional file 1: Figure S1. — The correlations of docking scores between Surflex and Vina, between PLANTS and Vina, and between PLANTS and Surflex, respectively. Figure S2. The 200ns MD trajectory of VP24. Figure S3. The binding mode of the top 20 ranked drugs against VP24. (a) Montelukast, (b) Indinavir, (c) Iloprost, (d) hSalmeterol Xinafoate, (e) Travoprost, (f) Latanoprost, (g) Remikiren, (h) Vitamin K1, (i) Mitoxantrone, (j) Labetalol hydrochloride, (k) Tafluprost, (l) Misoprostol, (m) Carboprost, (n) Fosinopril, (o) Benzylpenicilloyl Polylysine, (p) Bimatoprost, (q) Nebivolol, (r) Valrubicin, (s) Tamsulosin, (t) Mycophenolate Mofetil. Figure S4. The 3D binding mode of the top 20 ranked drugs on the target of VP24. (a) Montelukast, (b) Indinavir, (c) Iloprost, (d) hSalmeterol Xinafoate, (e) Travoprost, (f) Latanoprost, (g) Remikiren, (h) Vitamin K1, (i) Mitoxantrone, (j) Labetalol hydrochloride, (k) Tafluprost, (l) Misoprostol, (m) Carboprost, (n) Fosinopril, (o) Benzylpenicilloyl Polylysine, (p) Bimatoprost, (q) Nebivolol, (r) Valrubicin, (s) Tamsulosin, (t) Mycophenolate Mofetil. Figure S5. Structural quality assessment of the homology model of O’-2-MTase. (a) Verify3D score. The sphere in red color showed the residues of composing binding site. (b) Ramachandran Plot using PROCHECK. Figure S6. The predicted binding mode of drugs that are listed in Table 3 in 2’-O-MTase. The drugs in the panels are: (a) SAM, (b) aza-S-adenosyl-L-methionine, (c) Sinefungin, (d) A9145C, (e) Maraviroc, (f) Abacavir, (g) Telbivudine, and (h) Cidofovir. (DOCX 3178 kb) [file 12859_2016_941_MOESM1_ESM.docx]
